# Single Real Goal, Magnitude-Based Deceptive Path-Planning

**DOI:** 10.3390/e22010088

**Published:** 2020-01-10

**Authors:** Kai Xu, Yunxiu Zeng, Long Qin, Quanjun Yin

**Affiliations:** College of Systems Engineering, National University of Defense Technology, Changsha 410073, China; xukai09@nudt.edu.cn (K.X.); zengyunxiu@nudt.edu.cn (Y.Z.); qinlong@nudt.edu.cn (L.Q.)

**Keywords:** deception, path-planning, plan recognition, information entropy

## Abstract

Deceptive path-planning is the task of finding a path so as to minimize the probability of an observer (or a defender) identifying the observed agent’s final goal before the goal has been reached. It is one of the important approaches to solving real-world challenges, such as public security, strategic transportation, and logistics. Existing methods either cannot make full use of the entire environments’ information, or lack enough flexibility for balancing the path’s deceptivity and available moving resource. In this work, building on recent developments in probabilistic goal recognition, we formalized a single real goal magnitude-based deceptive path-planning problem followed by a mixed-integer programming based deceptive path maximization and generation method. The model helps to establish a computable foundation for any further imposition of different deception concepts or strategies, and broadens its applicability in many scenarios. Experimental results showed the effectiveness of our methods in deceptive path-planning compared to the existing one.

## 1. Introduction

Deceptive planning in an adversarial environment enables humans or AI agents to cover their real intentions or mislead an opponent’s situation awareness. This would be of great help to many real-world applications, such as deceptive network intrusion [1], robotic soccer competition [2], intelligence reconnaissance [3], real-time strategy games, privacy protection [4], important convoy escorting, strategic transportation, or even military operations. Deceptive path-planning is one of its representative tasks. Masters and Sardina [5,6] first elaborated this problem and proposed three basic metrics, extent, density, and magnitude, to define deception. Among them, magnitude corresponds to any quantified measures of path deception at each individual node or step and has not been talked about yet.

This paper mainly uses the *magnitude* metric to measure the path deceptivity. To compute its value, based on the classical model-based goal recognition approaches [7], our method inverts recognition results and further uses them in planning the deceptive path. Goal recognition evaluates the posterior P(G|O) over the possible goal set *G* given observation *O*; further, P(G|O) is inversely used to quantify different forms of deception defined over each individual node. Further, we exploit the recent finding stated in [8], which has also been applied in [5], that goal recognition in path-planning does not need refer to any other historical observation given the observed agent’s starting point and its current location. This finding enables that at any node, the posterior probability distribution upon all possible goals, along with the following deception values, could be precalculated at any time, and has nothing to do with the path taken to reach that node.

With the deception being defined upon each separate node or step, a deceptive path could be easily generated when we maximize deception along the path from the start to the end. Inspired by this intuition, the paper proposes a single real goal magnitude-based deceptive path-planning model, as well as a deceptive path generation model formed into a mixed-integer programming problem. The advantages of the model are generalized as follows: firstly, using magnitude to quantify deception at each node helps to establish a computable foundation for any further imposition of different deception strategies, and thus broadens the model’s applicability in other scenarios with different deception characteristics; secondly, differently from the previous method [5] where deception could only be exploited from two goals (the real goal and a specific bogus goal), our method fully takes advantage over all possible goals during the generation of deceptive path; lastly, a moving resource could be flexibly defined to serve the deceiver’s trade-off between deceptivity and resource.

The paper is organized as follows. Section 2 describes a single real goal magnitude-based deceptive path planning framework. Section 3 presents a single real goal magnitude-based deceptive path generation method formulated into a mixed-integer programming problem. We present several classical deceptive strategies which have been talked about under different names in many studies, along with a newly proposed one. Also, a cyclic deceptive path identification method is presented in order to handle cycles that may occur in the optimization results. We conclude with a case study on an 11 × 11 grid map, an empirical evaluation, and a conclusion.

## 2. Background and Related Work

This work lies at the intersection of two well-established and much-studied disciplines within computer Ssience: path-planning, which is the problem of finding a path through a graph or grid from a given start to a given goal [9,10,11]; and probabilistic goal recognition, which tries to infer an agent’s goals that are evaluated continuously in the form of a probabilistic distribution over goals, given some or all of the agent’s observed actions [7,12,13,14].

Grounded on the proposition that the order of goals ranked by likelihood can surely be inverted to describe their unlikelihood, the paper uses the results of the probabilistic goal recognition to plan a deceptive path using optimization method. Thus in this section, we first introduce the basic concepts, background, and applications of deception, especially the deception in path-planning. After that, we talk about the related works in goal or plan recognition, and review the recent developments of problem formulations, models, and algorithms applied in probabilistic goal recognition.

### 2.1. Deception and Deceptive Path-Planning

The deception problem is significant; it is common, and occurred frequently in human history [15]. As for its popularity, it is also a topic with a long history in computer science, particularly within the realms of artificial intelligence [16], non-cooperative game theory [3,17,18,19], and one of increasing relevance—in social robotics [20]. Deception is a key indicator for intelligence, as shown by a study investigating the role of working memory in verbal deception in children [21]. Intelligent agents, computer generated forces, or non-player characters who apply deceptive strategies are more realistic, challenging, and fun to play against [22] both in video games and serious training simulations. Furthermore, the potential use of deception has also been recognized in many multiagent scenarios, such as negotiation [23,24], multi-object auctioning [25], pursuit-evasion [26,27,28], and card games [29].

Defined by [30] and we quote here, deception is “the conscious, planned intrusion of an illusion seeking to alter a target’s perception of reality, replacing objective reality with perceived reality”. In a more dedicated definition [31], the deceptive tactics applied in the above applications could be further partitioned into two classes, denial (hiding key information) and deception (presenting misleading information). Tactics like masking, repackaging, dazzling, and red flagging are grouped in the denial type, while mimicking, inventing, decoying, and double play belong to the second one. These two patterns of deception with significant differences take turns at appearing in many studies, though the authors usually do not explicitly distinguish between them.

As a more focused area, research on the deceptive path appears in the literature under various guises. Jian et al. [32] tried to study the deception in path trajectories drawn by human subjects who had been asked beforehand to deceive an imaginary observer using a paper-and-pencil tests. Analyzed using both geographical and qualitative methods, the paper captured 38 recognizable characteristics, showing the existence of deception patterns and strategies in human behaviors, including *denial* and *deception*.

Hespanha et al. [17,18] and Root et al. [3] studied how deception could be used by rational players in the context of non-cooperative games. Hespanha [17] showed that, when one of the players can manipulate the information available to its opponents, deception can be used to increase the player’s payoff. Interestingly however, when the degree of possible manipulation is too high, deception becomes useless against the intelligent opponent, as the opponent makes the decision as if there are no observations at all. This exactly accords with the objective of the *denial* strategy. Using the same strategy but in a more practical case, Root et al. [3] studied the deceptive path generation applied in UAVs’ reconnaissance missions while under the opponent’s surveillance and fire threat. In a domain modeled as a graph, the system selects a ground path, and then constructs a set of flight plans that involve overflying not only that path but every edge capable of supporting military traffic. The execution of the paths renders observation meaningless: the defender must select from multiple routes, all with the same probability.

*Deception* strategy (presenting misleading information) arose in a path-planning-related experiment carried out by roboticists Shim and Arkin [33], inspired by the food-hoarding behavior of squirrels. Computerized robotic squirrels visit food caches and, if they believe themselves to be under surveillance, also visit false caches (where there is no food). On the basis of observed activity, a competitor decides which caches to raid and steals whatever food she finds. In tests, the deceptive robots kept their food significantly longer than non-deceptive robots, confirming the effectiveness of the strategy.

Recent innovative work on goal recognition design (GRD) [34,35,36,37,38] could be seen as an inverse problem to deceptive path-planning. Standing on the side of the observer, the GRD problem tries to reduce goal uncertainty and advance the correct recognition through redesigning the domain layout. To do so, they introduce a concept named “worst case distinctiveness (wcd), measuring the maximal length of a prefix of a plan an agent may take within a domain before its real goal has been revealed. At first, the wcd is calculated and minimized relying on three simplifying assumptions [34], one of which assumes that the agents are fully optimal. Thus, the type of deception they design against takes more of a form like the denial strategy.

Interestingly, following the continued research on the GRD problem where the original assumptions are gradually relaxed, another form of deception appears in the literature as well. When considering suboptimal paths, the authors of [35] focused on a *bounded nonoptimal* setting, where an agent was assumed to have a specified budget for diverting from an optimal path. Additionally, as they presented, it suits for situations where deceptive agents aim at achieving time-sensitive goals, with some flexibility in their schedule. This exactly is the condition for the deception strategy (presenting misleading information) to be applied. Though holding a different perspective, the GRD problem provides valuable insights to the study of deceptive path-planning.

The most recent work by Masters et al. [5] presents a model of deceptive path-planning, and establishes a solid ground for its future research. In their work, three measures—magnitude (at each step), density (number of steps), and extent (distance travelled)—are proposed to quantify path deception. Focusing particularly on extent, they introduce the notion of last deceptive point (LDP) and a novel way of measuring its location. Also, the paper explicitly applies the “denial” and “deception” strategies, termed by Masters as “simulation” (showing the false) and “dissimulation” (hiding the real), in planning the deceptive path. Still, the work [5] has several shortcomings to overcome. Firstly, as discussed in the last section, the LDP concept is narrowly defined and would lower the deception performance in certain situations. Also, their model loses sight of people’s needs in generating deceptive path with various path lengths, as resource constraints cannot be represented in their model. Lastly, though they have tried to enhance path deception by unifying “denial” and “deception”, e.g., additional refinements ($\pi_{3},\pi_{4}$ as in [5]) under the original dissimulation strategy, their model lacks the ability to combine the two or even more in one framework.

A closely related topic to deceptive path-planning is the deceptive or adversarial task planning. Braynov [39] presents a conceptual framework of planning and plan recognition as a deterministic full-information simultaneous-moves game and argues that rational players would play the Nash equilibrium in the game. The goal of the actor is to traverse an attack graph from a source to one of few targets and the observer can remove one of the edges in the attack graph per move. The approach does not describe the deception during the task planning and provides no experimental validation. Taking a synthetic domain inspired by a network security problem, Lisý [40] defines the adversarial goal recognition problem as an imperfect-information extensive-form game between the observer and the observed agent. In their work, a Monte-Carlo sampling approach is proposed to approximate the optimal solution and could stop at any time in the game.

The research on deception arises in many other studies which we mention here only briefly: in cyber security [41], privacy protection [4,42], and goal/plan obfuscation [43,44].

### 2.2. Probabilistic Goal Recognition

The goal recognition problem has been formulated and addressed in many ways, as a graph covering problem upon a plan graph [45], a parsing problem over grammar [46,47,48,49], a deductive and probabilistic inference task over a static or dynamic Bayesian network [12,50,51,52,53,54], and an inverse planning problem over planning models [7,55,56,57,58].

Among the approaches viewing the goal or plan recognition as an uncertainty problem, two formulations appear and solve the problem from different perspectives. One focuses on constructing a suitable library of plans or policies [51,52], while the other one takes the domain theory as an input and use planning algorithms to generate problem solutions [7,55]. The uncertainty problem is first addressed by the work [51], which phrases the plan recognition problem as the inference problem in a Bayesian network representing the process of executing the actor’s plan. It was followed by more work considering dynamic models for performing plan recognition online [47,48,59,60]. While that offers a coherent way of modeling and dealing with various sources of uncertainty in the plan execution model, the computational complexity and scalability of inference is the main issue, especially for dynamic models. In [52], Bui et al. proposed a framework for online probabilistic plan recognition based on the abstract hidden Markov model (AHMM), which is a stochastic model for representing the execution of a hierarchy of contingent plans (termed policies). Scalability in policy recognition in the AHMM, and the inference upon its transformed dynamic Bayesian network, are achieved by using an approximate inference scheme known as the Rao–Blackwellised particle filter [61].

Within that formulation, research continues to improve the model’s expressiveness and computational attractiveness, as in [12,54,62,63,64,65,66]. Also, recently machine learning methods including reinforcement learning [66], deep learning [67,68], and inverse reinforcement learning [69,70], have already been successfully applied to learning the agents’ decision models for goal recognition tasks. These efforts once again extend the usability of policy-based probabilistic goal recognition methods by constructing agents’ behavior models from real data.

Recently, the work [55,56] shows that plan recognition can be formulated and solved using off-the-shelf planners, followed by work considering suboptimality [7] and partial observability [57]. Not working over libraries but over the domain theory where a set G of possible goals is given, this generative approach solves the probabilistic goal recognition efficiently, provided that the probability of a goal is defined in terms of the cost difference of achieving the goal under two conditions: complying with the observations and not complying with them. By comparing cost differences across goals, a probability distribution is generated that conforms to the intuition: the lower the cost difference, the higher the probability.

Several works followed that approach [58,71,72,73,74] by using various automated planning techniques to analyze and solve goal or plan recognition problems. Its basic ideas have also been applied in new problems, such as goal recognition design [34] and deceptive path-planning [5]. The advantages of the latter formulation are twofold: one is that by using plenty of off-the-shelf model-based planners the approach scales up well, handling domains with hundred of actions and fluents quite efficiently; the other one lies in the fact that the model-based method has no concerns about the recognition of joint plans for achieving goal conjunctions. Joint plans come naturally in the generative approach from conjunctive goals, but harder to handle in library-based methods.

According to the relationships between the observer and the observed agent, the goal or plan recognition could be further divided into keyhole, intended, and adversarial types [1,75,76,77]. In keyhole plan recognition, the observed agent is indifferent to the fact that its plans are being observed and interpreted. The presence of a recognizer who is watching the activity of the planning agent does not affect the way he plans and acts [59,78]. Intended recognition arises, for example, in cooperative problem-solving and in understanding indirect speech acts. In these cases, recognizing the intentions of the agent allows us to provide assistance or respond appropriately [75]. In adversarial recognition, the observed agent is hostile to the observation of his actions and attempts to thwart the recognition [79,80].

## 3. The Single, Real Goal, Magnitude-based Deceptive Path-Planning

We first begin by formalizing path planning and goal recognition. We propose a rather simple but general model suitable for different deceptive path planning domains. We assume that there are two players: one is the evader (the observed agent); the other one is the defender (the observer). The evader chooses one goal from a set of possible goals and attempts to reach it, whereas the defender must correctly recognize which goal has been chosen. We assume that the outcomes of the action are deterministic and the model is fully observable to the evader and the defender, meaning that the environment and each others’ actions are visible to both players. In addition, the evader has several assumptions about the defender. Firstly, the defender does not know the decision-making process of the evader. Secondly, the defender operates under a fixed recognition algorithm.

The notion of a road network captures the intuition about all possible deceptive paths that can be planned against a set of goals.

**Definition** **1.** *A road network is a triplet RN=〈N,E,c〉, where:*

- *N is a non-empty set of nodes (or locations);*
- *E⊆N×N is a set of edges between nodes;*
- *$c:E\to R_{0}^{+}$ returns the length of each edge.*

Define the path π as a sequence of nodes through a road network, and $\pi^{i}$ as the *i*-th node in π. The cost of path π is the sum of length of all the edges in π, and we have $cost\left(\pi \right)=\sum_{i=0}^{k-1}c\left(\pi^{i},\pi^{i+1}\right)$. In its formal definition, the path-planning problem considers a set of goals; in our setting, however, we give a single-goal path-planning problem as follows.

**Definition** **2.** *The single-goal path-planning problem is a triplet SGPP=〈RN,s,g〉, where:*

- *RN=〈N,E,c〉 is the road network;*
- *s∈N is the source;*
- *g⊆N is the single goal/destination/target.*

The solution to the single-goal path-planning problem is a path which starts in its source *s* and ends in one single goal *g*. Denote $optc(n_{i},n_{j})$ as the cost of the optimal solution from the node $n_{i}$ to $n_{j}$.

**Definition** **3.** *The probabilistic goal recognition problem is a tuple PGR=〈RN,s,G,O,Prob〉, where:*

- *RN=〈N,E,c〉 is the road network;*
- *s∈N is the source;*
- *$G\;=\;\left\{g_{r}\right\}\cup G_{b}$ is the possible goal set, where $g_{r}$ is the single real goal, and $G_{b}$ is the set of bogus goals;*
- *$O\;=\;o_{1},\ldots ,o_{\left|O\right|}\in N$ is the observation sequence;*
- *Prob are the prior probabilities upon the possible goal set G.*

A solution to the probabilistic goal recognition is a conditional probability distribution P(G|O) across *G* given observation sequence *O*, with each two neighboring observations $o_{i},o_{i+1}\in O$ not needing to be temporally adjacent. The quality of solution, or we could say that of the goal recognition algorithm, could be reflected from whether it has $P\left(g_{r}|O\right)\geq P\left(g|O\right)$ for all $g\in G_{b}$. Further, in many real-world applications with high risk and interest, only $P\left(g_{r}|O\right)\geq P\left(g|O\right)$ in PGR’s solutions may not be enough.

In this paper, we take a concept *costdif* which was applied early in [7] to reflect the goals’ uncertainties at each node or location. Specifically, it captures the intuition that *costdif* has an inverse correlation to goals’ posterior probabilities; that is

**Theorem** **1.** *$P\left(g|O\right)>P\left(g^{\prime }|O\right)$ if and only if costdif(s,g,O)$<\mathrm{costdif}(s,g^{\prime },O)$.*

The *costdif*(s,g,O) first defined in [7] represents the cost difference between the optimal cost optc(s,g,O) of plans that accords with observation and $optc(s,g,\bar{O})$ that is not. The posterior probability distribution upon goal set *G* could be computed using:

(1) $$P\left(G|O\right)\;=\;\alpha \frac{e^{-\beta X}}{1+e^{-\beta X}}$$

where X=costdif(G,O), α is a normalizing constant across goals and β is a positive constant which captures a “soft rationality” assumption.

As we have talked about in the last section, Masters and Sardina [8] proved that in path-planning domain, goal recognition needs not refer to any other historical observation given the observed agent’s source and current location. In other words, only the final observation in the sequence that matters:

(2) $${\mathrm{costdif}}_{\mathrm{MS}}\left(s,g,n\right)\;=\;\mathrm{optc}\left(n,g\right)-\mathrm{optc}\left(s,g\right)$$

where $n\;=\;O^{\left|O\right|}$ is the last observed location of the evader. Like in Theorem 1, we have:

**Theorem** **2.** *$P\left(g|n\right)>P\left(g^{\prime }|n\right)$ if and only if costdif(s,g,n)$<\mathrm{costdif}(s,g^{\prime },n)$.*

Both Theorem 2 and Equation (2) enable that at any node, we could precalculate the posterior probability distribution upon all possible goals in an offline manner.

**Definition** **4.** *The single real goal deceptive path-planning problem is a tuple $SRGDPP\;=\;\langle RN,s,G,g_{r},P\rangle$, where:*

- *RN=〈N,E,c〉 is the road network;*
- *s∈N is the source;*
- *$G\;=\;\left\{g_{r}\right\}\cup G_{b}$ is the possible goal set;*
- *$g_{r}$ is the single real goal;*
- *P(G|O) denotes the posterior probability distribution upon G given O.*

Specifically, in this paper we could replace P(G|O) as P(G|n) where *n* is the last node in that sequence. *P* stands for the observation model of the defender. We assume that *G* consists of all the possible goals (e.g., multiple highway entrances, airports, or harbors in a city), including a single real goal $g_{r}$ and other bogus goals $g_{b}\in G_{b}$. The evader starts from *s* and moves towards $g_{r}$, while during the process, making use of both the possible goal sets *G* as well as the defender’s observation model *P* to plan a deceptive path. Corresponding to the single-goal path-planning problem, this work considers a single real goal situation and leaves the multiple-real-goals setting to the future research. We assume that the evader has different deception strategies on planning the path.

**Definition** **5.** *The evader’s deceptive strategy is a function $F:P\left(G|i\right)\to {\mathrm{mag}}_{i}$, where P(G|i) is the set of probability distributions at the node i∈N of road network RN over the possible goals G, ${\mathrm{mag}}_{i}$ is the deceptive magnitude at node i.*

Different strategies evaluate a different deceptive magnitude at each node. Later, we introduce strategies in detail. Further, we present a single, real goal, magnitude-based deceptive path planning model as follows:

**Definition** **6.** *The single real goal, magnitude-based deceptive path-planning is a triplet SRGMDPP=〈SRGDPP,F,R〉, where:*

- *$\mathrm{SRGDPP}\;=\;\langle RN,s,G,g_{r},P\rangle$ is the single real goal deceptive path-planning problem;*
- *$F:P\left(G|i\right)\to {\mathrm{mag}}_{i}$ returns the deception magnitude value assigned to each node;*
- *R is the total amount of distance allowed for the deceiver traversing $s-g_{r}$ path.*

Apart from the elements in SRGDPP problem, the SRGMDPP adds a deceptive strategy function that maps the probabilistic goal recognition results into quantified path deception at each individual node. Also, a explicit presentation of total resource constraint enables the evader to flexibly adjust its deceptive path plan according to the time (or fuel) constraints in practice. We say that SRGMDPP provides a computable foundation for further impositions of different deception concepts or strategies, and broadens the model’s applicability in other scenarios.

## 4. The Single Real Goal Magnitude-Based Deception Maximization

Inspired by the intuition that a deceptive path could be generated when maximizing deception along the *s*–$g_{r}$ path, and following the above definitions, we present its mathematical formulation as follows.

### 4.1. Model Formulation

Denote i,j∈N as the nodes and k=(i,j)∈E the edge in the road network. The edge sets FS(i) and RS(i) represent the set of edges directed out of and into the node *i*. Assign the same magnitude value $mag_{i}$ of node *i* to values $mag_{k}^{\prime }$ of all the edges *k* in the set RS(i), where $mag_{k}^{\prime }$ is the deceptive magnitude associated with edges. The formulation is:

$$\left[\mathrm{SRGMDM}-P\right]\;\max_{x\in X}\sum_{k\in E}\left(1+mag_{k}^{\prime }\right)\cdot c_{k}\cdot x_{k}$$

(3) $$\sum_{k\in FS(i)}\;\;\;x_{k}\;-\;\;\;\sum_{k\in RS(i)}\;\;\;x_{k}\;\;=\;\;\left\{\;\;\begin{array}{cc} 1 & \;\mathrm{for}\;i=s\\ 0 & \;\forall i\in N⧹\{s,g_{r}\}\\ -1 & \;\mathrm{for}\;i=g_{r} \end{array}\right.$$

(4) $$x_{k}\geq 0,\;\forall k\in E$$

where $c_{k}$ (the vector form c) is the cost of traversing edge *k*; $x_{k}$ (the vector form x) is the integer variable controlling whether the evader traverses the edge *k* or not, $x_{k}=1$ if the edge *k* is traversed, or else, $x_{k}=0$. The solution $X=\{x\in {\left\{0,1\right\}}^{\left|E\right|}|c^{T}x\leq R\}$. Equation (3) is the flow-balance constraint, which guides the evader to leave *s* and reach $g_{r}$ and guarantees the nodes *i* in the set $N⧹\{s,g_{r}\}$ to be visited and leaved at the same number of times. Soon, we give three kinds of measures of deceptivity: two were first talked about in [5]; one is proposed based on them.

### 4.2. Deceptive Strategy

**Definition** **7.** *The simulation strategy $F_{sim}\left(P\left(G|i\right)\right)\;=\;\max_{g_{b}\in G⧹\left\{g_{r}\right\}}P\left(g_{b}|i\right)-P\left(g_{r}|i\right)$.*

The simulation strategy measures the amount by which a false goal dominates the real goal. And the greatest deception occurs when there are the greatest dominance. $F_{sim}$ drives the defender believes that the evader’s target is one bogus goal (“showing the false”), and thus deploys resource to the wrong location.

**Definition** **8.** *The dissimulation strategy $F_{dissim}\left(P\left(G|i\right)\right)\;=\;\sum_{g\in G}P\left(g|n\right)\times \log_{2}\left(P\left(g|n\right)\right)$.*

As a popular method, this type of deception (or we say the degree of ambiguity) is defined using the Shannon’s entropy, which is usually used to describe information uncertainty. By taking advantage of the defender’s observation model, the $F_{dissim}$ strategy plans a path where different goals share similar posterior (“hiding the real”), and thus making the defender confused about where to deploy its resource. Instead, the defender will make decisions, as there are no observations at all, as discussed about in [17], though it may also guess correctly. Also, this strategy could prove its effectiveness in applications with high risk or interest, as without certainty it is itself a danger. Usually, compared to a simulation, this strategy consumes less resource.

However, both concepts of deception reflect only a fraction of what it means to be deceptive. The difference lies in the extent to which people want to deceive the observer. According to the strength of intention protection desire, usually these two strategies could be balanced in human behaviors. Thus the paper proposes a more flexible weighted combination strategy.

**Definition** **9.** *The combination strategy $F_{combi}\left(P\left(G|i\right)\right)\;=\;\omega_{1}\cdot F_{sim}+\omega_{2}\cdot F_{dissim}$, where $\omega_{1}+\omega_{2}\;=\;1$.*

We quantify that aspect of deception as a linear combination of the simulation and dissimulation. If we deceive successfully using the combination strategy, the defender would either be led to the false goal (when $\omega_{1}$ is larger), or choose randomly in a set of possible goals (when $\omega_{2}$ is larger).

**Figure 1:**
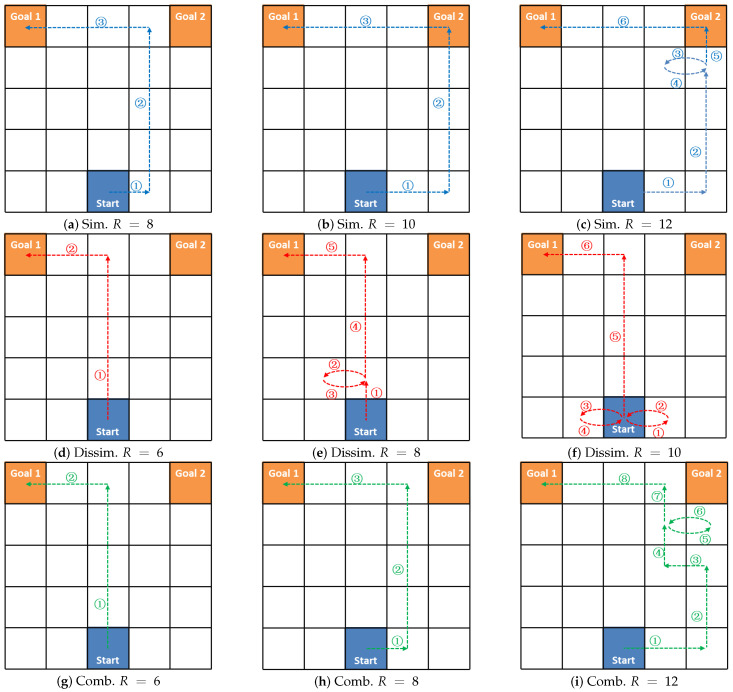
The deceptive path generated using the MDM model under different resource constraint *R*.

Take the example, as shown in Figure 1, where the paper presents groups of deceptive paths optimized using SRGMDM-P under different resource constraints. Specifically, Figure 1a–c follows simulation (Sim.) strategy, Figure 1d–f accords with dissimulation (Dissim.), while paths in Figure 1g–i respect the combined one (Comb.). Several trends need to be noted here. When resources are insufficient for the agent planning the most deceptive way, the model returns a less deceptive version; e.g., the path in Figure 1a with R=8 is less deceptive than the ones in Figure 1b or Figure 1c. In this case, it is the same as the one in Figure 1h following the combined strategy. A cyclic path appears when the deceiver is given additional resource, as we can see in Figure 1c,e,f,i. This satisfies the needs of those agents who either are time-sensitive to other cooperative missions or just have abundant resources. Paths generated following the combined strategy would be more like those under dissimulation when resources are inadequate (e.g., Figure 1d,g, but transform more like the ones under simulations with more resources coming in (refer to Figure 1c,i).

### 4.3. Cyclic Deceptive Path Identification

The result of the SRGMDM model is the optimized variable *X*, whose nonzero elements comprise the set $E_{X}$ of edges that would be traversed by the evader. From the edge set $E_{X}$, a solution road network ${\mathrm{RN}}_{X}\;=\;\langle N_{X},E_{X},c_{X}\rangle$ could be further generated, where $N_{X}$ is a non-empty set of nodes that appears in the edges from $E_{X}$, and $c_{X}$ denotes the length of original edge.

**Figure 2:**
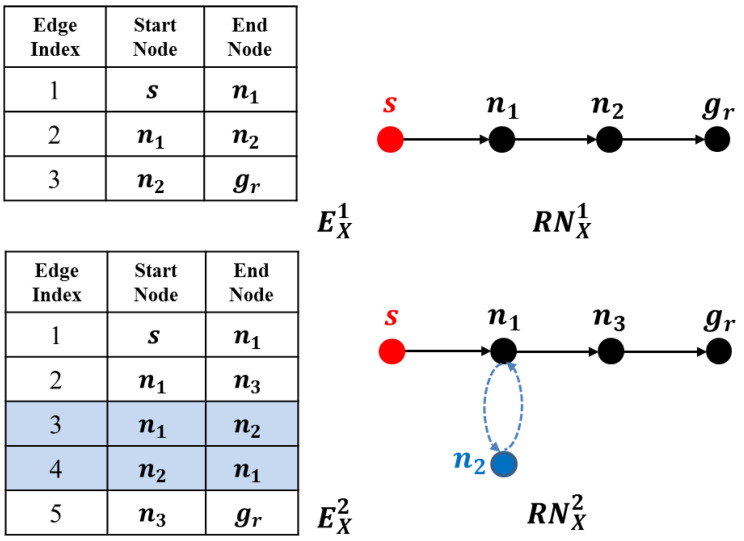
The deceptive paths with and without cycles on two solution road networks, ${\mathrm{RN}}_{X}^{1}$ and ${\mathrm{RN}}_{X}^{2}$.

When no circles exist in the deceptive path, for example, in the optimal path planning problem, given start position *s* and destination $g_{r}$, the agent’s moving trajectories could be easily sketched out by linking edges from end to end. For example, in the first case shown in Figure 2, where $E_{X}=\left\{\left(s,n_{1}\right),\left(n_{1},n_{2}\right),\left(n_{2},g_{r}\right)\right\}$, the corresponding path is $s\to n_{1}\to n_{2}\to g_{r}$. However, when cyclic paths occur, as in the case below, where $E_{X}\;=\;\left\{\left(s,n_{1}\right),\left(n_{1},n_{3}\right),\left(n_{1},n_{2}\right),\left(n_{2},n_{1}\right),\left(n_{3},g_{r}\right)\right\}$, this simple method may fail as the edges 3 and 4 (edges $\left(n_{1},n_{2}\right)$ and $\left(n_{2},n_{1}\right)$ marked in blue) will not be traversed by the evader.

An intuitive way to generate cyclic path is to try exhaust every possible combination that could cover all edges in $E_{X}$. For example, we could change the orders of edge numbers 2 and 3 in the above case, and get the path $s\to n_{1}\to n_{2}\to n_{1}\to n_{3}\to g_{r}$. However, it wiould become more complicated when much more circles exist in one path, as is the case in Figure 1f.

Before introducing our method for identifying the cyclic deceptive path from $E_{X}$, the paper first considers the situation when cyclic path could appear in a solution road network.

**Definition** **10.** *In a solution road network ${\mathrm{RN}}_{X}\;=\;\langle N_{X},E_{X},c_{X}\rangle$, the cyclic path appears when it satisfies either one of the following conditions:*

- *|FS(s)|−|RS(s)|=1, and |FS(s)|>1;*
- *$\exists i\in N_{X}⧹\left\{s,g_{r}\right\},|\mathrm{FS}\left(i\right)|\;=\;|\mathrm{RS}\left(i\right)|>1$.*

*The nodes following the above conditions are grouped by the set $N_{X}^{\prime }$.*

The edge sets FS(i) and RS(i) represent the sets of edges directed out of and into the node *i*. The nodes in $N_{X}^{\prime }$ locate on cyclic trajectories that may appear in the whole path.

**Theorem** **3.** *In the solution road network ${\mathrm{RN}}_{X}\;=\;\langle N_{X},E_{X},c_{X}\rangle$, consider the case where |FS(i)|=2 for $i\in N_{X}$; there will always exist one edge j∈FS(i) whose ending node has a way of returning back to i.*

**Proof.**  Assume to the contrary that all the ending nodes of edges in FS(i) have no way returning back to *i*; then, according to the constraints in Equation (3), there must be edges starting from the other node (denoted as *j*) that direct to node *i*. This in turn means that node *j* cannot be reached from *i* and *j* must be in earlier positions than *i* of the whole path. Then, consider two cases: (1) when i=s, node *i* itself locates in the first place before any other nodes; (2) for $i\in N_{X}^{\prime }⧹\left\{s\right\}$, when the node *i* is reached, a trajectory starting from *s* must have been drawn out already. Then, *j* is either on this trajectory, or it is not. When *j* is on, it cannot contribute one more edge into *i*. When it is not, two trajectories, (s,…,j,i) and the other one (s,…,i) with no *j*, coexist, and that contradicts the task of generating only one path from $E_{X}$. □

The above theorem could be easily extend to more general situations.

**Theorem** **4.** *For the node $i\in N_{X}$, there will always exist |FS(i)|−1 numbers of distinct paths returning back to i from the ending nodes of edges j∈FS(i).*

The above observations assure us that when confronted with nodes $i\in N_{X}^{\prime }$, we could first find passable ways starting from FS(i) to *i*, add these edges into the ordered edge set, and continuously do these two steps until no elements in FS(i) could return back to *i*. Relying on Theorems 3 and 4, we present the *cyclic-path-identify* algorithm (whose pseudocode is given in Algorithm 1).
**Algorithm 1:***Cyclic-path-identify algorithm*
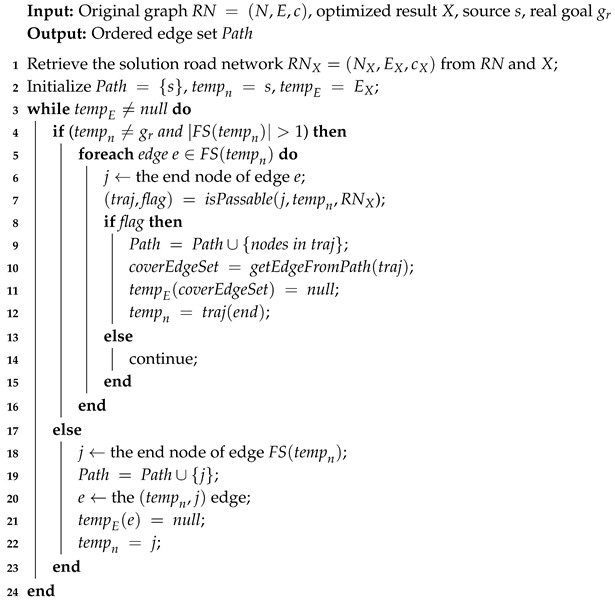


Algorithm 1 shows how to identify the cyclic path from the optimization result *X*, given the original road network RN=(N,E,c) and the source *s*. We first retrieve the solution road network $RN_{X}=\left(N_{X},E_{X},c_{X}\right)$ from $RN_{X}$ and *X*, and initialize an ordered edge set Path (Lines 1–2). Only when all edges in $E_{X}$ have been added into Path, the process (Lines 4–23) can stop. For nodes not located on cyclic trajectory (Situation 1), they could be simply added to Path using an end-to-end style (Lines 18–22). While for those on the cycles (Situation 2), judged by conditions we have presented above, the method generates all the trajectories traj starting from end nodes *j* of $FS({\mathrm{temp}}_{n})$, and adds the covered edges coverEdgeSet in trajectories into Path. This process (Lines 4–16) relies on Theorem 3 and 4, as they two guarantee that when facing cycles, there will always exist |FS(i)|−1 numbers of distinct paths returning back to *i* from the ending nodes of edges j∈FS(i). Through linking these distinct paths to Path in |FS(i)|−1 times, finally the problem degrades from Situation 2 to Situation 1. Two additional steps are required for the process under both conditions: (1) deleting the covered edges from $temp_{E}$; (2) directing ${\mathrm{temp}}_{n}$ to the new node. The A* path-finding algorithm is used in the isPassable function at line 7, where traj is a generated path starting from the node *j* and ending at $temp_{n}$, flag signals whether a path could be found.

## 5. Experiments

**Figure 3:**
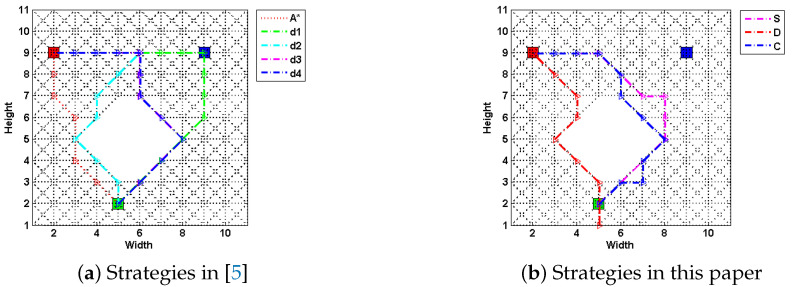
The deceptive path following the four strategies proposed in [5], and three in this paper upon a simple 11 × 11 grid-based road network, where the green square is the source, red is the single real goal $g_{r}$, and blue is the bogus one. An optimal path generated by A* is given as a comparison. An underlying assumption is that the agent would traverse the road network at a pace of one grid a time step.

**Figure 4:**
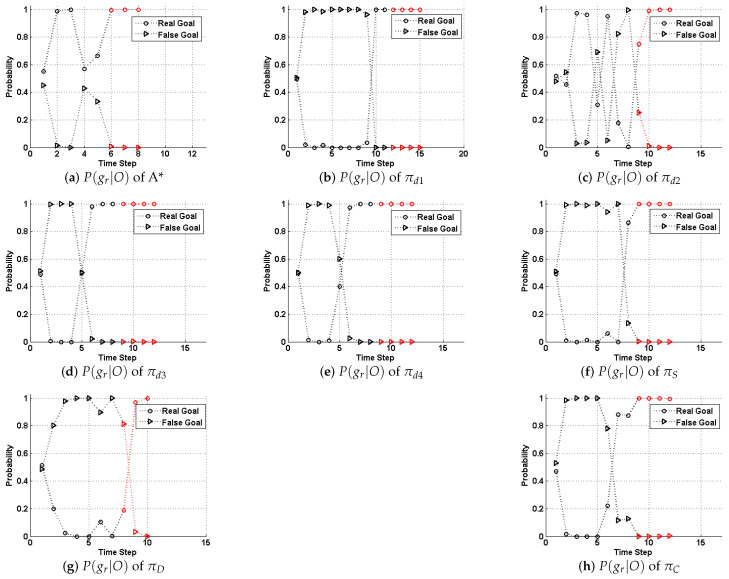
Probabilistic goal recognition results of the paths planned following the strategies shown in Figure 3. The section of $P(g_{r}|O)$ whose path after the last deceptive point is marked red.

In this section, we compare the performance of the model and strategies proposed by this paper with those (strategies d1 to d4) by [5]. First we present a case study upon a fixed 11 × 11 grid map to elaborate our method’s performance, as shown in Figure 3. Methods are compared by metrics including generation time, path costs and their deceptivity, as shown in Table 1. Also, a probabilistic goal recognition method applied in [54] generates a posterior probability distribution P(G|O) across possible goal set *G* given *O*, as shown in Figure 4. We set the weights of *combination* strategy to $\omega_{1}=\omega_{1}=0.5$ to have a balanced performance between “showing the false” and “hiding the real.” The computation of the SRGMDM was formulated into a mixed-integer programming and solved using the solvers of CPLEX 11.5 and YALMIP toolbox of MATLAB 2012 [81].

Specifically, in Figure 3a, we show four strategies in [5] along with an optimal path-planning case (using A* algorithm) as a comparison. Strategy d1 (the green dashed line), which is treated as a simplest “showing the false” strategy in [35], first takes an optimal path towards a bogus goal (marked as a blue square). Computationally inexpensive, this achieves a strongly deceptive path (referring to Table 1). However, the cost of its path is usually very high and, although it initially deceives both human and automated observers, reaching but not stopping at the bogus goal (which is the blue square in this case) immediately signals to a human that it is not the real goal. This is also evident as in Figure 4b, where $P(g_{r}|O)$ immediately rises high when the evader moves away from the bogus goal after time Step 8.

Path d2 (the magenta dashed line) is regarded as a simplest “hiding the truth,” by which the evader takes an optimal path $\pi_{d2}$ directly from the source *s* to the last deceptive point (LDP), and then on to $g_{r}$. As we have talked about in the related works, the LDP is a concept and a node defined in [5], in which the authors believe beyond this point all nodes in the path are truthful (a truthful node is one at which $P\left(g_{r}|O\right)>P\left(g_{b}|O\right)$ for all $g_{b}\in G_{b}$). In Figure 4, we mark the part of path behind the LDP as red).

It might be more deceptive to a human observer until later in the path than $\pi_{d1}$ above. However, $\pi_{d2}$ is only weakly deceptive; that is, truthful steps are likely to occur before the LDP without additional checks and balances. As we could find in Table 1, the goal recognition cannot really tell which one is the right one. While in Figure 4c, this trend is clearer than the posterior probability of the real goal, that of the bogus goal, alternatively, becomes the larger one.

Strategies d3 and d4 are two refinements of d2. A path following the strategy d3 can be assembled using a modified heuristic so that, while still targeting the LDP, whenever there is a choice of routes, it favors the bogus goal, increasing its likelihood of remaining deceptive. d4 prunes all truthful nodes during the search from source *s* to the LDP, thereby requiring more time compared to the other three. However, upon this simple case study, there does not exist too much difference between d3 and d4.

In Figure 3b, we show the paths generated using the SRGMDM model, where the deceptivity is measured by its magnitude at each step, following the simulation “S,” dissimulation “D,” and combination “C” strategies defined in this paper. Currently, we constrain the path cost of all three to no more than 13.07, the same as d2, d3 and d4, but smaller than d1. From the results shown in Table 1, paths following *S* and *C* fully deceive the defender at each 10%, 25%, etc., of their path length prior to the LDP, while *D* strategy generates a cyclic path at the beginning (marked as red dashed line in Figure 3b) and loses its deceptivity in the last two-fifths of path length. This is reasonable, as both d2 and *D* only try to make the defender not know the real goal, though it may still guess correctly. Compared to strongly-deceptive strategies d1 and *S*, “showing the false” is not their primary focus, and thus in most situations they are more efficient in terms of cost.

As for the results of strategies *S*, *D*, and *C* shown in Figure 3, we could see that, before the LDP, all strategies deceive the defender well. For example in Figure 4f, the $P(g_{r}|O)$ is continuously dominated by that of the bogus goal until time step 7. While after the LDP, the goal recognizer successfully identifies the real goal behind the evader’s misleading actions. It also should be noted that, the goal recognition method we use here is more sensitive to observations, as it captures the evader’s real goal several steps ahead of the LDP in cases like Figure 4b,d,e,h.

The case study shows the effectiveness of our method in planning deceptive paths. Also, paths generated using SRGMDM do not rely on the so-called LDP as a transit point before moving towards the real goal, and the trajectories are more flexible, as shown in Figure 3. As LDP is calculated only according to the real goal $g_{r}$ and the bogus goal which is the closest to $g_{r}$, as stated in [5]; our method instead fully takes advantage of the global information (including all possible goals) during the deceptive path-planning. Besides, the method’s flexibility also shows, as cyclic deceptive paths would be generated whenever the moving resource is abundant.

**Figure 5:**
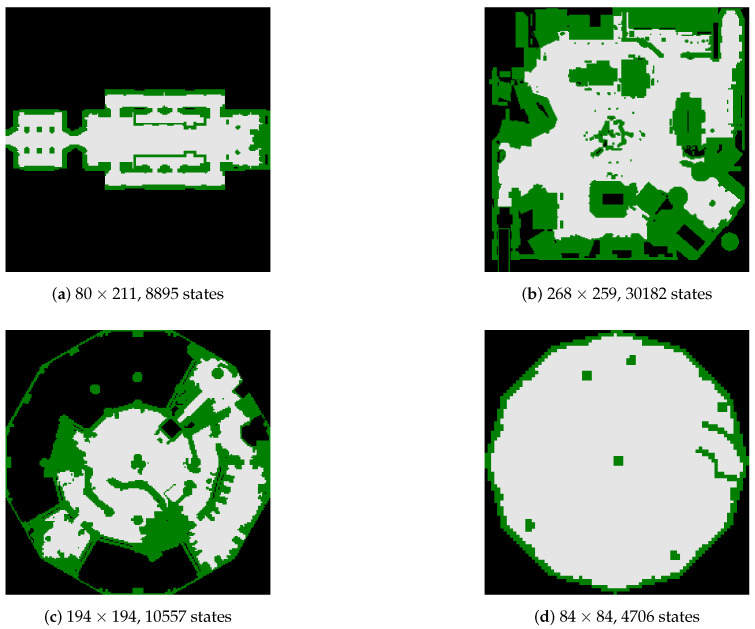
Four large-scale 2D grid maps from the moving-AI benchmarks.

In order to have a more thorough comparison between different methods and strategies, in the following experiments, we conduct an empirical evaluation over a problem set which is generated upon the grid-based maps from the moving-AI benchmarks [82]. The source and the real goal are already available in the benchmark. Bogus goals are added randomly at different locations. Figure 5 shows four 2D grid map examples that are selected for tests from the benchmarks.

As with the above cast study, in each case we generated one optimal path using the standard A* and seven deceptive paths, each corresponding to a different deceptive strategy. Using probabilistic goal recognition and under the assumption that priors for all goals are equal, we calculated probabilities at intervals to confirm/assess deceptive magnitude. As to evaluating traces with different lengths, the paper normalizes traces with different lengths into *t*$\left(t=1,2,\ldots ,N_{\mathrm{stage}}\right)$ intervals. $N_{\mathrm{stage}}$ is the total number of intervals that we want to partition.

We got the statistical results using the metric of *F-measure*, which is frequently used to measure the overall accuracy of the recognizer [14] and is computed as:

(5) $$\mathrm{precision}\;=\;\frac{1}{\left|G\right|}\sum_{m=1}^{\left|G\right|}\frac{{\mathrm{TP}}_{m}}{{\mathrm{TI}}_{m}},\mathrm{recall}\;=\;\frac{1}{\left|G\right|}\sum_{m=1}^{\left|G\right|}\frac{{\mathrm{TP}}_{m}}{{\mathrm{TT}}_{m}}$$

(6) $$F-\mathrm{measure}\;=\;\frac{2\cdot \mathrm{precision}\cdot \mathrm{recall}}{\mathrm{precision}+\mathrm{recall}}$$

where |G| is the number of possible goals; ${\mathrm{TP}}_{m}$, ${\mathrm{TI}}_{m}$, and ${\mathrm{TT}}_{m}$ are the true positives, total of true labels, and the total of inferred labels for class $g_{m}\in G$ respectively. The value of *F-measure* will be between 0 and 1, and a higher value means a better confidence of recognizing the right goal while a less deceptive strategy. Thus, the lower value, the more deceptive.

**Figure 6:**
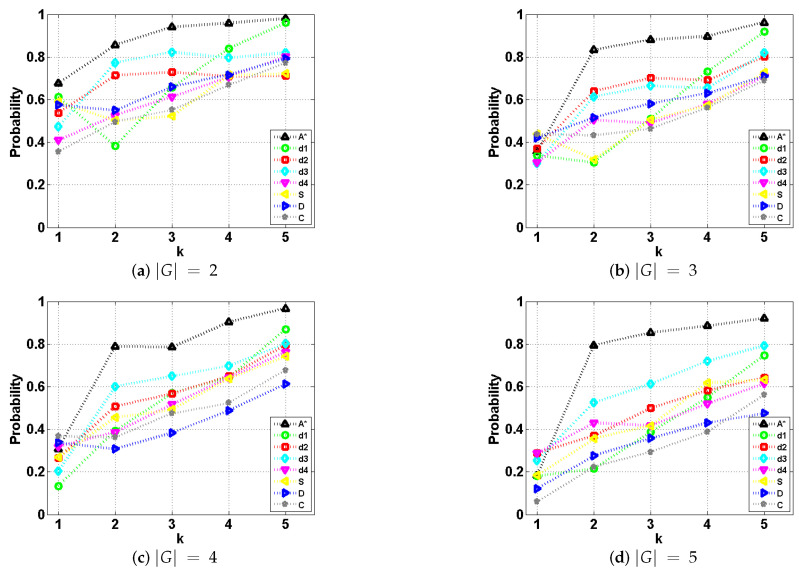
The *F-measure* of the goal recognizer with deceptive paths generated using different strategies, at each interval *t* under different goal settings ($N_{\mathrm{stage}}\;=\;5$).

The results are shown in Figure 6. The weights of *combination* strategy were set the same as in the above case study. The resource constraints of paths following simulation, dissimulation, and combination were the same as those of paths using d2 strategy. As a general trend, we could find that the path deceptivity gradually decreased as more observation coming in. A* provides a performance baseline for comparisons with other strategies. Among all the strategies, the *F-measure* of A* increases greater than 80% early from interval t=2, indicating the disappearance of path deceptivity. As stated in their work [5], strategy d2 and d3 are weakly deceptive, while d1 and d4 are strongly deceptive. Similarly, in our results, apart from A*, d2 and d3 generate the least deceptive paths in most situations. As for d1, different from their findings however, it performs the best in the first half (interval t≤2) of deceptive path. However, as its paths do not stop at the closest bogus goal, the goal recognizer immediately changes its belief to the real goal; thus, when t≥4, the performance of d1 deteriorates the fastest among all the others. This is because the goal recognition algorithm we use considers more history information during the whole process while the method in [5] is not. From the results, we admit that d4 is the most deceptive strategy compared to d1, d2, and d3.

Now, we compare the performance of d4 with that of *simulation*, *dissimulation* and *combination*. Firstly, we could find that in all situations, the *simulation* strategy always has a similar (when |G|=3,4,5) or even better performance (when |G|=2) compared to d4.

Interestingly, for *dissimulation* and *combination* strategies, paths become more deceptive as the number of possible goals |G| increases. Specifically, *dissimulation* is better than d4 when |G|>3. It continuously drags the value of *F-measure* down from 0.8 to 0.7, 0.6 and below 0.5 when |G| increases from 2 to 5. Though *dissimulation* fails d4 when |G|≤3, this strategy’s advantage lies in its ability in solving multiple goals situation.

*Combination* is strictly better than d4 in all cases. As a balanced strategy between *simulation* and *dissimulation*, two concepts of deception are both embedded in one strategy, and thus the merits of the two enable the *combination* strategy to outperform d4 in different situations. For example, it has a similar performance to *simulation* when |G|≤3 and *dissimulation* when |G|>3. With other concepts of deception suited for different problem domains or tasks coming in, *combination* or a similar balanced strategy using different weight settings, along with our magnitude-based deception maximization model could be of great help in planning suitable paths.

The experiments prove the effectiveness of our method in making full use of environment information, compared to the work in [5] that narrowly focuses on finding the tipping point (LDP) where the probability of the real goal becomes equal to that of some other goal. Overall, the results shown in Figure 6 prove that our single real goal magnitude-based deceptive path-planning has a decent, better, and more flexible performance.

## 6. Conclusions

In this paper, we formalise deception as it applies to path-planning and present strategies for the generation of deceptive paths. The paper propose a single, real goal, magnitude-based deception maximization model which establishes a computable foundation for any further imposition of different deception strategies, and broadens the model’s applicability in many scenarios. Different from the previous method [5] where deception could only be exploited from two goals (the real goal and a specific bogus goal), our method fully takes advantage of all possible goals during the generation of deceptive path. Also, the moving resource can be flexibly defined to serve the deceiver’s trade-off between deceptivity and resource. As for the deceptive strategy, taking advantage of the model’s flexibility, we propose a weighted combination strategy based on the original simulation and dissimulation. In general, our method shows much more flexibility and generality compared to the existing work from many aspects.

Though this work is quite suggestive, there are still many open problems that could be researched in the future. Firstly, the paper assumes that the defender is naive and rational. It is naive because the defender does not expect to be deceived, and thus deploys no counter plans or tactics. Its rationality is inherited from the goal recognition paradigm. If it is not, on one hand it may use machine learning approaches, e.g., the work in [70], to adapt its goal recognition model, or it would take more active counter behaviors; e.g., in the public security domain restricting the evader’s actions at certain locations by using patrolling vehicles [34]. Further, it could be formulated in terms of a zero-sum game in which both players are trying to act optimally [83]. At this time, it would be more complicated to model these interactions between the two.

Secondly, the current model assumes that the environment and deceiver’s actions are fully observable to the goal recognizer or the observer. However, for partially observable situations where its actions could be masked and behavioral patterns be covered, we would expect many more tactics could be available for the evader. Also as current formulation only considers one real goal setting, this work could be further extended to multiple real goal settings and thus transforming the problem into non-linear path-planning, which would be a much more valuable scenario where the evader have more than one targets.

Lastly, we believe that the concepts of deception, along with its models, could also be applied in other domains, such as classical planning using a plan library, etc. Similarly, a probabilistic plan recognition algorithm could then be used to evaluate the magnitude of deception, and a newly proposed optimization model with resource constraint to maximize the plan deceptivity.

## Figures and Tables

**Table 1:** The details of traces generated using different strategies, and the deceptivity (**1** = deceptive, **0** = correct prediction) of path when tested at 10%, 25%, etc., of their path lengths *prior to* the last deceptive point (LDP). (**St** = strategy, **C** = path cost, **T** = generation time ($\times 10^{-3}s$)).

St	C	T	10%	25%	50%	75%	99%
A*	8.24	0.69	1	1	0	0	0
d1	15.66	2.37	1	1	1	1	1
d2	13.07	4.93	1	0	0	0	1
d3	13.07	3.81	1	1	1	1	1
d4	13.07	66.08	1	1	1	1	1
S	13.07	849.81	1	1	1	1	1
D	11.07	220.79	1	1	1	0	0
C	13.07	145.23	1	1	1	1	1

## Abbreviations

The following abbreviations are used in this manuscript:

AI	Artificial Intelligence
UAV	Unmanned Aerial Vehicle
LDP	Last Deceptive Point
AHMM	Abstract Hidden Markov Models
GRD	Goal Recognition Design
wcd	worst-case distinctiveness
RN	Road Network
SGPP	Single Goal Path-Planning
PGR	Probabilistic Goal Recognition
SRGDPP	Single Real Goal Deceptive Path-Planning
SRGMDPP	Single Real Goal Magnitude-based Deceptive Path-Planning
SRGMDM	Single Real Goal Magnitude-based Deception Maximization
